# Intentional Self-Harm Among US Veterans With Traumatic Brain Injury or Posttraumatic Stress Disorder: Retrospective Cohort Study From 2008 to 2017

**DOI:** 10.2196/42803

**Published:** 2023-07-24

**Authors:** Bhanu Pratap Singh Rawat, Joel Reisman, Terri K Pogoda, Weisong Liu, Subendhu Rongali, Robert H Aseltine Jr, Kun Chen, Jack Tsai, Dan Berlowitz, Hong Yu, Kathleen F Carlson

**Affiliations:** 1 Manning College of Information and Computer Sciences University of Massachusetts Amherst Amherst, MA United States; 2 Center for Healthcare Organization & Implementation Research VA Boston Healthcare System Bedford, MA United States; 3 Center for Healthcare Organization & Implementation Research VA Boston Healthcare System Boston, MA United States; 4 Boston University School of Public Health Boston, MA United States; 5 Center of Biomedical and Health Research in Data Sciences University of Massachusetts Lowell Lowell, MA United States; 6 Division of Behavioral Sciences and Community Health UConn Health Farmington, CT United States; 7 Department of Statistics University of Connecticut Storrs, CT United States; 8 Center to Improve Veteran Involvement in Care VA Portland Health Care System Portland, OR United States; 9 Oregon Health & Science University-Portland State University School of Public Health Portland, OR United States

**Keywords:** self-harm, suicide, suicide attempt, suicidal ideation, veteran, suicidal, brain injury, trauma, posttraumatic stress disorder, PTSD, big data, prevalence, correlation, risk factor, traumatic brain injury, TBI

## Abstract

**Background:**

Veterans with a history of traumatic brain injury (TBI) and/or posttraumatic stress disorder (PTSD) may be at increased risk of suicide attempts and other forms of intentional self-harm as compared to veterans without TBI or PTSD.

**Objective:**

Using administrative data from the US Veterans Health Administration (VHA), we studied associations between TBI and PTSD diagnoses, and subsequent diagnoses of intentional self-harm among US veterans who used VHA health care between 2008 and 2017.

**Methods:**

All veterans with encounters or hospitalizations for intentional self-harm were assigned “index dates” corresponding to the date of the first related visit; among those without intentional self-harm, we randomly selected a date from among the veteran’s health care encounters to match the distribution of case index dates over the 10-year period. We then examined the prevalence of TBI and PTSD diagnoses within the 5-year period prior to veterans’ index dates. TBI, PTSD, and intentional self-harm were identified using International Classification of Diseases diagnosis and external cause of injury codes from inpatient and outpatient VHA encounters. We stratified analyses by veterans’ average yearly VHA utilization in the 5-year period before their index date (low, medium, or high). Variations in prevalence and odds of intentional self-harm diagnoses were compared by veterans’ prior TBI and PTSD diagnosis status (TBI only, PTSD only, and comorbid TBI/PTSD) for each VHA utilization stratum. Multivariable models adjusted for age, sex, race, ethnicity, marital status, Department of Veterans Affairs service-connection status, and Charlson Comorbidity Index scores.

**Results:**

About 6.7 million veterans with at least two VHA visits in the 5-year period before their index dates were included in the analyses; 86,644 had at least one intentional self-harm diagnosis during the study period. During the periods prior to veterans’ index dates, 93,866 were diagnosed with TBI only; 892,420 with PTSD only; and 102,549 with comorbid TBI/PTSD. Across all three VHA utilization strata, the prevalence of intentional self-harm diagnoses was higher among veterans diagnosed with TBI, PTSD, or TBI/PTSD than among veterans with neither diagnosis. The observed difference was most pronounced among veterans in the high VHA utilization stratum. The prevalence of intentional self-harm was six times higher among those with comorbid TBI/PTSD (6778/58,295, 11.63%) than among veterans with neither TBI nor PTSD (21,979/1,144,991, 1.92%). Adjusted odds ratios suggested that, after accounting for potential confounders, veterans with TBI, PTSD, or comorbid TBI/PTSD had higher odds of self-harm compared to veterans without these diagnoses. Among veterans with high VHA utilization, those with comorbid TBI/PTSD were 4.26 (95% CI 4.15-4.38) times more likely to receive diagnoses for intentional self-harm than veterans with neither diagnosis. This pattern was similar for veterans with low and medium VHA utilization.

**Conclusions:**

Veterans with TBI and/or PTSD diagnoses, compared to those with neither diagnosis, were substantially more likely to be subsequently diagnosed with intentional self-harm between 2008 and 2017. These associations were most pronounced among veterans who used VHA health care most frequently. These findings suggest a need for suicide prevention efforts targeted at veterans with these diagnoses.

## Introduction

For decades, suicide has been one of the leading causes of death [1,2] and a major health concern among veterans in the United States [3]. The most recent Department of Veterans Affairs (VA) Office of Mental Health and Suicide Prevention’s National Veteran Suicide Prevention Annual Report stated that the annual suicide rate for veterans (31.6 per 100,000) was twice the rate of that for nonveteran US adults (16.8 per 100,000) [4].

Suicide prevention is a top clinical priority for the VA and its nationally integrated health care system, overseen by the Veterans Health Administration (VHA) [4]. Central to this effort is the identification of individuals at risk for suicide or suicidal behavior [5,6]. A substantial body of research has used electronic health record data to identify individuals likely to make suicide attempts and other forms of intentional self-harm [7-10]. Research has shown that adults and youths with a history of nonfatal self-harm are at a markedly elevated risk of suicide [11-13].

Other important risk factors for suicidal behavior include sociodemographic, psychological, and physical health characteristics [14-17]. In particular, two health conditions relatively common among veterans—traumatic brain injury (TBI) and posttraumatic stress disorder (PTSD)—have been identified in multiple studies as risk factors for suicide [2,18-20]. For example, Bullman and Kang [21] reported that Vietnam veterans with PTSD were at elevated risk of suicide death when compared with those without PTSD. Another study by Jakupcak et al [22] observed similar patterns for post-9/11 veterans, in which veterans with PTSD were reported to have a four times greater likelihood of suicidal ideation as compared to veterans without PTSD. In a 2011 study, Brenner et al [18] reported that PTSD was associated with 2.8 times greater odds of a documented suicide attempt compared to veterans without PTSD; the authors suggested that future analyses compare those with and without comorbid TBI history. In another study, Brenner et al [20] conducted an analysis to examine the associations between TBI diagnosis and suicide among individuals receiving care at VHA and reported that veterans with TBI were 1.55 times more likely to complete suicide as compared to those without TBI. The mechanisms by which TBI or PTSD lead to an elevated risk of suicide or suicidal behaviors are not well understood, but the associations are consistent with constructs of risk described in the interpersonal theory of suicide (ie, thwarted belongingness, perceived burdensomeness, and acquired capability) [23]. Notably, individuals with TBI or PTSD often have co-occurring physical health (eg, chronic pain), mental health (eg, depression and anxiety), and behavioral conditions (eg, substance use disorder) that can lead to functional limitations and other vulnerabilities (eg, unemployment and homelessness) [24-28], which together or individually, can elevate the factors comprising each of these constructs [29].

Previous studies examining associations between TBI and PTSD diagnoses and suicidal behavior have mostly been conducted with relatively small or select samples or with veterans from a single war era [2,18,19]. These studies have suggested that TBI and PTSD are important risk factors for suicidal behavior. Moreover, previous studies have tended to only analyze the associations of TBI [30] and PTSD [31] separately with suicidal behavior and have not examined the potential interactive effects of comorbid TBI and PTSD.

In this study, we analyzed data from approximately 6.7 million veterans who received VHA health care between 2008 and 2017 to examine population-level variations in the prevalence of intentional self-harm among those with or without prior TBI and PTSD diagnosis. We focused on suicide attempts as well as other intentional self-harm events because previous research [11,12] has shown that adults and youths were at a markedly elevated risk of suicide completion after nonfatal self-harm events. Our analysis was stratified by level of VHA utilization to account for differential access to VHA care and the opportunity to receive the diagnoses of interest. Our objectives were to evaluate variation in the prevalence of intentional self-harm by veterans’ prior TBI and PTSD diagnosis status across different VHA utilization levels, and to examine the odds of veterans’ intentional self-harm by prior TBI and PTSD diagnosis status across different VHA utilization levels while controlling for potentially confounding variables. The overarching goal of this work is to inform suicide and other intentional self-harm prevention efforts.

## Methods

### Ethics Approval

This study was approved by the VA Bedford Healthcare System Institutional Review Board. We identified 6,693,275 veterans who visited a VHA facility at least twice during the 10-year period between January 1, 2008, and December 31, 2017. We extracted veterans’ demographic and health care data from the VHA Corporate Data Warehouse (CDW) database. All preprocessing and analyses were conducted using the VA Informatics and Computing Infrastructure (VINCI) accessed from Bedford, Virginia.

### Dependent Variable

This study defined intentional self-harm as suicide attempts or other self-harm using diagnosis codes specified for those behaviors supplemented with additional evidence that an injury incurred by a veteran was due to self-harm. Suicide attempt or intentional self-harm was defined as a health care encounter (outpatient visit, including urgent care, or hospitalization) that met any of three conditions: presence of a code that explicitly indicated suicide attempt or intentional self-harm, presence of codes identifying suicidal ideation *and* injury or poisoning assigned for the same encounter, or presence of codes identifying injury or poisoning *and* diagnoses for a mental disorder assigned for the same encounter. Patrick et al [32] used this definition based on diagnosis and external cause of injury codes from the *International Classification of Diseases, Ninth Revision, Clinical Modification (ICD-9-CM*) manual. For this study, we derived a set of *International Statistical Classification of Diseases, Tenth Revision* (*ICD-10*) codes that corresponded with the *ICD-9* codes used by Patrick et al [32]. The algorithms and ICD codes used are provided in Table S1 in Multimedia Appendix 1.

### Independent Variable

For veterans with one or more intentional self-harm events, we chose the first coded incident of intentional self-harm as the “index date.” For veterans with no intentional self-harm events, we randomly selected a date from among the veterans’ encounters to match the distribution of dates among those with self-harm events in the 10-year period. Each veteran was classified into one of four mutually exclusive groups based on the presence or absence of TBI and PTSD diagnoses during the 5 years prior to the veteran’s index date:

- No TBI or PTSD diagnoses
- TBI diagnosis only
- PTSD diagnosis only
- Comorbid TBI/PTSD diagnoses

The codes used to identify both TBI and PTSD are included in Table S2 in Multimedia Appendix 1.

To account for “informed presence” bias based on the varying opportunity to receive the diagnosis codes of interest arising from differential health care utilization [33], we classified veterans into 3 groups according to the average annual VHA health care visits in the 5-year period before their “index date.” The *low* VHA utilization group included those at the 50th percentile and below (≤6.6 visits per year), the *medium* utilization group included those above the 50th but below and at the 75th percentiles (>6.6 and ≤15.6 visits), and the *high* utilization group included those above the 75th percentile (>15.6 visits). Analyses were stratified by VHA utilization group.

### Covariates

Veterans’ age, sex, race, ethnicity, marital status, and VA service-connection status—a measure of military service–related disability assigned by the Veterans Benefits Administration—were extracted from the CDW. Age was categorized as <30, 30-44, 45-69, or ≥70 years. Sex was categorized as a binary variable, while race was categorized as White, Black or African American, Asian, Native Hawaiian or other Pacific Islander, American Indian or Alaska Native, or other and ethnicity as Hispanic versus non-Hispanic. Marital status was categorized as single, married, divorced, or widowed, and VA service-connection as no service-connection, service-connected <50%, or service-connected ≥50%. We also computed the Charlson Comorbidity Index (CCI) scores using diagnoses from inpatient and outpatient visits, and categorized veterans as having low (<5), medium (5-15), or high (>15) Charlson Comorbidity scores [34].

### Analyses

We calculated the prevalence of veterans’ intentional self-harm (yes/no) according to TBI and PTSD status for each VHA health care utilization group. We modeled the likelihood of intentional self-harm among veterans by TBI and PTSD status using logistic regression to estimate odds ratios (ORs) and their corresponding 95% CIs. Adjusted ORs (AORs) were calculated using two multivariable logistic regression models: minimally adjusted models that controlled for age, sex, and race, and fully adjusted models that controlled for age, sex, race, ethnicity, marital status, VA service-connection status, and CCI scores.

## Results

### Overview

Among the 6,693,275 veterans who used VHA health care between 2008 and 2017 at least twice, 93.1% (n=6,231,400) were coded as male and 6.9% (n=461,874) as female; 76.74% (n=5,136,343) were coded as being White, 15.85% (n=1,060,951) Black or African American, 0.98% (n=65,402) Asian, 0.84% (n=56,167) American Indian or Alaska Native, 0.96% (n=63,943) Native Hawaiian or other Pacific Islander, and 4.64% (n=310,469) as other (Table 1). More than one-third of veterans (n=2,318,144, 34.63%) were service-connected at 50% or higher. The VHA health care utilization strata had the following composition of veterans: *low* (n=3,327,615, 49.72%); *medium* (n=1,679,954, 25.09%), and *high* (n=1,686,066, 25.19%). Overall, we identified 86,644 (1.29%) veterans with one or more encounters involving intentional self-harm. TBI and PTSD were relatively prevalent among VHA users, with 1.4% (n=93,866) having been diagnosed with TBI only, 13.33% (n=892,420) with PTSD only, and 1.53% (n=102,549) with comorbid TBI/PTSD.

**Table 1:** Prevalence of intentional self-harm by veterans’ sociodemographic and health variables, stratified by VHA utilization.

			Low VHA^a^ utilization					Medium VHA utilization				High VHA utilization		
			Self-harm, n (%)		Total veterans, n		Self-harm, n (%)			Total veterans, n	Self-harm, n (%)			Total veterans, n
**Age (years)**														
	<30	2256 (0.93)		243,318		3007 (5.76)			52,241		4150 (15.85)		26,188	
	30-44	2712 (0.54)		497,756		4841 (2.61)			185,421		12,119 (8.17)		148,312	
	45-69	5107 (0.35)		1,472,935		9838 (1.15)			854,474		36,067 (3.63)		993,893	
	≥70	669 (0.06)		1,113,606		1228 (0.21)			587,458		4650 (0.9)		517,673	
**Sex**														
	Female	1031 (0.49)		212,124		2097 (1.85)			113,257		6772 (4.96)		136,493	
	Male	9713 (0.31)		3,115,490		16,817 (1.07)			1,566,337		50,214 (3.24)		1,549,573	
**Race**														
	White	7958 (0.31)		2,608,591		14,277 (1.1)			1,292,525		42,159 (3.41)		1,235,227	
	Black or African American	1801 (0.38)		469,890		3114 (1.18)			263,253		10,856 (3.31)		327,808	
	Asian	170 (0.42)		40,872		176 (1.24)			14,187		363 (3.51)		10,343	
	American Indian or Alaska Native	129 (0.46)		27,919		231 (1.71)			13,482		734 (4.97)		14,766	
	Native Hawaiian or other Pacific Islander	140 (0.45)		31,152		256 (1.58)			16,157		503 (3.02)		16,634	
	Other	546 (0.37)		149,191		860 (1.08)			79,990		2371 (2.92)		81,288	
**Ethnicity**														
	Hispanic	705 (0.4)		175,414		1272 (1.35)			94,218		3667 (3.56)		102,962	
	Not Hispanic	10,039 (0.31)		3,152,201		17,642 (1.11)			1,585,376		53,319 (3.36)		1,583,104	
**Marital status**														
	Single	2437 (0.57)		427,381		3838 (2.04)			188,239		11,463 (5.55)		206,491	
	Married	3839 (0.2)		1,940,210		6572 (0.71)			928,639		17,178 (2.11)		814,864	
	Widowed	377 (0.15)		253,548		755 (0.56)			135,848		2767 (2.05)		134,697	
	Divorced	4007 (0.59)		677,616		7663 (1.81)			422,514		25,495 (4.83)		527,684	
	Unknown	84 (0.29)		28,860		86 (1.98)			4354		83 (3.56)		2330	
**Veterans Affairs service-connection status**														
	No service-connection	4141 (0.25)		1,659,561		6184 (0.81)			759,889		16,890 (2.79)		605,966	
	Service-connection <50%	1806 (0.24)		751,492		2940 (0.88)			333,972		7918 (3.0)		264,251	
	Service-connection ≥50%	4797 (0.52)		916,562		9790 (1.67)			585,733		32,178 (3.94)		815,849	
**Charlson Comorbidity Index score**														
	≤5	7889 (0.38)		2,092,840		12,078 (1.52)			792,553		26,207 (4.72)		555,414	
	5-15	2787 (0.23)		1,221,470		6662 (0.77)			869,826		29,407 (2.72)		1,079,467	
	≥15	68 (0.51)		13,305		174 (1.01)			17,215		1372 (2.68)		51,185	

^a^VHA: Veterans Health Administration.

### Prevalence of Intentional Self-Harm by TBI and PTSD Diagnosis Status

The prevalence of intentional self-harm by TBI and PTSD diagnosis status is presented in Table 2, stratified by *low*, *medium*, and *high* VHA utilization groups.

Between 2008 and 2017, the overall prevalence of intentional self-harm was highest for veterans in the *high* VHA utilization group (56,986/1,743,052, 3.38%). In this stratum, the prevalence of intentional self-harm was highest for veterans in the comorbid TBI/PTSD diagnosis group (6778/58,295, 11.63%) and lowest among those with no TBI or PTSD diagnosis (21,979/1,144,991, 1.92%). Those in the TBI only and PTSD only diagnosis groups also had elevated prevalence levels, at 5.21% (2335/44,833) and 5.91% (25,894/437,947), respectively. This pattern of elevated prevalence was similar for veterans in the *medium* and *low* VHA utilization groups; however, the disparity between diagnosis groups in the *medium* and *low* VHA utilization groups was considerably lower than in the *high* VHA utilization group. Across all three VHA utilization groups, the prevalence of self-harm diagnosis among veterans with PTSD only was consistently higher by about 0.7% than those in the TBI only diagnosis group.

**Table 2:** Prevalence of intentional self-harm by veterans’ TBI and PTSD diagnosis status, stratified by Veterans Health Administration utilization.

				Intentional self-harm		
				Yes		No
Veteran diagnosis status, n				86,644		6,606,631
**Low health care utilization (less than 50th percentile), n (%)**						
	**TBI^a^ and PTSD^b^ diagnoses**					
		Neither	6792 (0.22)		3,065,664 (99.78)	
		TBI only	215 (0.84)		25,409 (99.16)	
		PTSD only	3407 (1.6)		209,468 (98.40)	
		Comorbid TBI/PTSD	330 (1.98)		16,330 (98.02)	
		Total	10,744 (0.32)		3,316,871 (99.68)	
**Medium health care utilization (50th-75th percentile), n (%)**						
	**TBI and PTSD diagnoses**					
		Neither	9466 (0.68)		1,377,527 (99.32)	
		TBI only	558 (2.38)		22,851 (97.62)	
		PTSD only	7548 (3.12)		234,050 (96.88)	
		Comorbid TBI/PTSD	1342 (4.86)		26,252 (95.14)	
		Total	18,914 (1.13)		1,660,680 (98.77)	
**High health care utilization (75th percentile and higher), n (%)**						
	**TBI and PTSD diagnoses**					
		Neither	21,979 (1.92)		1,123,012 (98.08)	
		TBI only	2335 (5.21)		42,498 (94.79)	
		PTSD only	25,894 (5.91)		412,053 (94.09)	
		Comorbid TBI/PTSD	6778 (11.63)		51,517 (88.37)	
		Total	56,986 (3.38)		1,629,080 (96.62)	

^a^TBI: traumatic brain injury.

^b^PTSD: posttraumtic stress disorder.

### Odds of Intentional Self-Harm by TBI and PTSD Diagnosis Status

Table 3 presents ORs and AORs based on bivariable and multivariable logistic regression models, again stratified by VHA utilization group. (ORs for model covariates are provided in Table S3 in Multimedia Appendix 1.)

Among veterans in the *high* VHA utilization group, and compared to veterans with no TBI or PTSD diagnoses, those with comorbid TBI/PTSD had the highest odds of intentional self-harm (OR 6.72, 95% CI 6.54-6.90), followed by those in the PTSD only group (OR 3.21, 95% CI 3.16-3.26) and then those in the TBI only group (OR 2.81, 95% CI 2.69-2.93). These patterns were similar in the fully adjusted model, where the AORs for comorbid TBI/PTSD group were the highest (AOR 4.26, 95% CI 4.15-4.38), followed by the PTSD only group (AOR 2.90, 95% CI 2.85-2.95) and then by the TBI only group (OR 2.44, 95% CI 2.34-2.55).

Among veterans in the *medium* VHA utilization group, the odds of intentional self-harm were highest among those with comorbid TBI/PTSD (OR 7.42, 95% CI 7.01-7.85), followed by the PTSD only group (OR 4.68, 95% CI 4.54-4.82) and then the TBI only group (OR 3.54, 95% CI 3.25-3.85). This pattern changed slightly when potential confounders were added to the models: AORs using the fully adjusted model were highest among those with PTSD only (AOR 3.23, 95% CI 3.14-3.33) and lowest among those with TBI only (AOR 2.32, 95% CI 2.13-2.53). The AOR for the comorbid TBI/PTSD group decreased from 7.42 (95% CI 7.01-7.85) to 3.17 (95% CI 2.99-3.36) after accounting for all available confounding variables.

Among veterans in the *low* VHA utilization group, the odds of intentional self-harm were also highest among those with comorbid TBI/PTSD (OR 9.05, 95% CI 8.10-10.11), followed by the PTSD only group (OR 7.33, 95% CI 7.04-7.64) and then the TBI only group (OR 3.79, 95% CI 3.31-4.34). Similar to the *medium* VHA utilization group, the pattern changed when potential confounders were added to the fully adjusted models: AORs for the PTSD only group (AOR 5.14, 95% CI 4.93-5.35) were highest, closely followed by the comorbid TBI/PTSD group (AOR 4.83, 95% CI 4.32-5.40) and then the TBI only group (AOR 2.48, 95% CI 2.17-2.84).

**Table 3:** Odds of intentional self-harm by veterans’ TBI and PTSD diagnosis status, stratified by Veterans Health Administration utilization.

Veteran diagnosis status				Logistic regression				
				Bivariable, OR^a^ (95% CI)		Multivariable, AOR^b^ (95% CI)		
				Model		Minimally adjusted model^c^		Fully adjusted model^d^
**Low health care utilization (less than 50th percentile)**								
	**TBI^e^ and PTSD^f^ diagnoses**							
		Neither	Reference		Reference		Reference	
		TBI only	3.79 (3.31-4.34)		2.60 (2.27-2.98)		2.48 (2.17-2.84)	
		PTSD only	7.33 (7.04-7.64)		5.22 (5.01-5.44)		5.14 (4.93-5.35)	
		Comorbid TBI/PTSD	9.05 (8.10-10.11)		4.85 (4.34-5.42)		4.83 (4.32-5.40)	
**Medium health care utilization (50th-75th percentile)**								
	**TBI and PTSD diagnoses**							
		Neither	Reference		Reference		Reference	
		TBI only	3.54 (3.25-3.85)		2.33 (2.14-2.54)		2.32 (2.13-2.53)	
		PTSD only	4.68 (4.54-4.82)		2.91 (2.83-3.00)		3.23 (3.14-3.33)	
		Comorbid TBI/PTSD	7.42 (7.01-7.85)		2.81 (2.65-2.97)		3.17 (2.99-3.36)	
**High health care utilization (75th percentile and higher)**								
	**TBI and PTSD diagnoses**							
		Neither	Reference		Reference		Reference	
		TBI only	2.81 (2.69-2.93)		2.50 (2.40-2.61)		2.44 (2.34-2.55)	
		PTSD only	3.21 (3.16-3.26)		2.35 (2.31-2.39)		2.90 (2.85-2.95)	
		Comorbid TBI/PTSD	6.72 (6.54-6.90)		3.40 (3.31-3.49)		4.26 (4.15-4.38)	

^a^OR: odds ratio.

^b^AOR: adjusted odds ratio.

^c^Minimally adjusted model included age, sex, and race.

^d^Fully adjusted model included age, sex, race, ethnicity, marital status, Veterans Affairs service-connection status, and Charlson Comorbidity Index scores.

^e^TBI: traumatic brain injury.

^f^PTSD: posttraumtic stress disorder.

## Discussion

Our analysis of approximately 6.7 million VHA-using veterans found that those with a documented diagnosis of TBI or PTSD were significantly more likely to have subsequent VHA documentation of a suicide attempt or other form of intentional self-harm relative to those with neither diagnosis between 2008 and 2017. For all three VHA utilization groups, the odds of intentional self-harm for veterans in the comorbid TBI/PTSD group were more than three times as high as those with neither condition, even after controlling for multiple potential confounders. For those in the PTSD only group, the adjusted odds of intentional self-harm were approximately three times that of those with neither condition. For the TBI only group, the adjusted odds of intentional self-harm were nearly double relative to those with neither condition. For all three VHA utilization groups, the prevalence of intentional self-harm for veterans in the comorbid TBI/PTSD group was higher than in the TBI only and PTSD only groups.

For the *medium* and *low* VHA utilization groups, adjusted odds were highest for the PTSD only group closely followed by the comorbid TBI/PTSD group. However, for the *high* VHA utilization group, adjusted odds for the comorbid TBI/PTSD group were consistently higher than those of the PTSD only group. Previous studies have suggested that symptom severity may play an important role in this association [35,36]. Our study did not consider symptom severity because this measure is difficult to ascertain in a large administrative data set such as the VHA’s CDW. Future research should consider how TBI and PTSD symptom severity drive functional outcomes and, in turn, may lead to intentional self-harm, as addressing functional limitations may be an important focus for prevention programs.

Psychological and functional impairments can be common for veterans with co-occurring PTSD and TBI [37]. PTSD is a known contributor to disability [38], resulting in more than US $40 billion in costs related to direct health care and unemployment among military populations [39]. Despite experiencing long-term symptoms of PTSD and the availability of various therapies, many individuals with PTSD wait years to decades before seeking professional treatment [40,41]. Patients may be skeptical toward therapy [42] and believe treatment to be ineffective, harmful [43], and only for extreme problems. Some may also believe treatment involves a loss of control or autonomy, or as something for people who are “weak,” “crazy,” or “incompetent” [40]. Some individuals with PTSD do not seek help at all, as only approximately one-quarter of individuals with PTSD symptoms receive mental health services [44,45]. A significant number of veterans drop out of their PTSD treatment altogether due to reasons such as work interference, stigma related to the ailment, confidentiality concerns, and perceived treatment ineffectiveness, thus contributing to a population of veterans with unresolved symptoms related to PTSD [46,47].

Similarly, veterans with TBI, regardless of severity, may have a variety of co-occurring psychological and physical health challenges for years following injury [48], including affective, cognitive, somatosensory, and vestibular postconcussive symptoms [49]. Other impairments, especially documented for post-9/11 veterans, may include social/family dysfunction, unemployment, and general difficulty with community reintegration [25]. Individuals with TBI also may not be completely aware of their limitations [50] and, therefore, unable to fully engage in treatment [51]. Given the observed difficulties of veterans with documented PTSD, TBI, or both, VHA suicide prevention services should increase efforts to educate veterans on the importance and nature of therapeutic options to better engage at-risk veterans with VA health care services.

Veterans with TBI or PTSD also face different challenges in seeking treatment such as problems with access, sociocultural environment, past medical experiences, and illness burden [42,45,52]. Access barriers are mainly organizational, due to lack of knowledge about VHA, and logistical, and include time-consuming and complex enrollment processes in VHA, veterans not knowing which VHA services they are eligible for, and time- and distance-related expenses [53]. VHA has made extensive efforts to mitigate these logistical problems, including the implementation of the community care program, which allows veterans to seek care in the community if VHA facilities are unable to furnish services or if long travel distances or appointment wait times pose a burden [54].

A study performed by McCarthy et al [5] suggests that the inclusion of the Recovery Engagement and Coordination for Health–Veterans Enhanced Treatment (REACH VET) program at the VHA has resulted in reduced prevalence of nonfatal suicide attempts along with other greater treatment engagement and care provided to veterans. The algorithm used in REACH VET to identify individuals in the facilities’ top 0.1% suicide risk tiers uses TBI and PTSD diagnoses as variables among the 381 measures used for prediction [6,17]. Our study strengthens the case for using TBI and PTSD as risk factors for suicide risk prediction in the algorithm. Our findings can further help in the identification of high suicide risk veterans by flagging patients with comorbid TBI/PTSD diagnosis using the already available identifiers used by REACH VET [2,18,55].

There are several limitations to take into account in the context of these results. First, this study only included veterans who received health care in VHA settings. Motives for veterans not seeking VHA care are complex, and thus these veterans likely have different TBI-, PTSD-, and intentional self-harm–related profiles. Second, this study relied on diagnoses in VHA administrative data to identify both TBI and PTSD diagnosis status and intentional self-harm outcomes. The *high* VHA utilization group, while comprising only 25.19% (1,686,066/6,693,275) of the study population, comprised 65.77% (56,986/86,644) of those with detected suicide attempts and other forms of intentional self-harm, 47.76% (44,833/93,866) of the TBI only group, 49.07% (437,947/892,420) of the PTSD only group, and 56.85% (58,295/102,549) of the comorbid TBI/PTSD group. It is likely that underdiagnosis was present [56]. For example, veterans within the *low* and *medium* VHA utilization groups may have experienced TBI, PTSD, or both but received care for them outside the VHA or not at all. Such misclassification of true exposure or outcomes status can bias results. However, we observed similar patterns across all three levels of VHA health care utilization, suggesting that results may not be explained entirely by informed presence bias. Third, our study used a broad range of diagnosis codes for intentional self-harm, per Patrick et al [32], to potentially decrease the likelihood that a true self-harm event would be missed (eg, if only suicide attempt codes were used). Future work that further examines and establishes the validity of more ICD codes to detect intentional self-harm would strengthen this line of research. Fourth, the analysis represented TBI and PTSD simply as present or absent according to diagnosis codes. A more refined analysis would incorporate the nature of trauma exposure, symptom severity, barriers to care, and social risk factors. Fifth, our work studied the associations of different TBI and PTSD diagnosis groups with intentional self-harm while adjusting for age, sex, race, ethnicity, marital status, VA service-connection status, and different comorbidities. This analysis did not directly examine the effects of these covariates on intentional self-harm. Further in-depth analysis is required to understand the associations between these sociodemographic and health variables and intentional self-harm while controlling for confounder sets relevant to those specific associations.

Our study adds to the literature on associations of TBI and PTSD diagnoses with intentional self-harm. It provides generalized results across a large veteran population of varied demographics and VHA utilization. Our findings indicate that veterans with a TBI and/or PTSD diagnosis are more likely to have an intentional self-harm event than veterans without either of these diagnoses. These results may contribute to VHA clinical decision-making regarding the frequency and intensity of follow-up or treatment for veterans with PTSD and TBI diagnoses.

Future research on the risk of intentional self-harm among veterans with TBI and/or PTSD can benefit from both qualitative and quantitative efforts. Qualitatively exploring veterans’ experiences and perceptions of health and functional status, as well as facilitators and barriers to health care services, can help to better identify health and access needs. Quantitatively exploring TBI and PTSD severity based on the nature of trauma exposure, current or previous treatment, and the intensity of posttraumatic symptoms can provide more granular associations between TBI and PTSD diagnosis and intentional self-harm. These efforts may help to inform clinicians in targeting preventative and treatment strategies toward a high-risk veteran cohort.

This study examined 6.7 million veteran VHA users and found that those with TBI and/or PTSD diagnoses were more likely to have documented intentional self-harm compared to veterans with neither diagnosis. Veterans with TBI, PTSD, or comorbid TBI/PTSD should be considered high-priority recipients of VA suicide prevention services and may need special engagement efforts given various access and treatment barriers experienced among those with these conditions.

## Appendix

Multimedia Appendix 1 Supplementary tables.

## Abbreviations

AOR: adjusted odds ratio
CCI: Charlson Comorbidity Index
CDW: Corporate Data Warehouse
ICD-10: International Classification of Diseases, Tenth Revision
ICD-9-CM: International Classification of Diseases, Ninth Revision, Clinical Modification
OR: odds ratio
PTSD: posttraumatic stress disorder
REACH VET: Recovery Engagement and Coordination for Health–Veterans Enhanced Treatment
TBI: traumatic brain injury
VA: Veterans Affairs
VHA: Veterans Health Administration
VINCI: VA Informatics and Computing Infrastructure
