# Supplementary material for: Intentional Self-Harm Among US Veterans With Traumatic Brain Injury or Posttraumatic Stress Disorder: Retrospective Cohort Study From 2008 to 2017
Source: JMIR Public Health Surveill. 2023 Jul 24;9:e42803. doi: 10.2196/42803 (PMC10407646; doi:10.2196/42803)
Supplement: Multimedia Appendix 1 [file publichealth_v9i1e42803_app1.docx]

**Supplemental Table 1**: ICD-9 and ICD-10 clinical modification (CM) diagnosis and external cause of injury codes used for identification of intentional self-harm.

| **Self-harm** | |
| --- | --- |
| **Rationale** | **ICD-9-CM Codes** |
| Suicide Attempt | E950-E958 |
| Suicide Ideation plus Injury or Poisoning | At same visit:  V62.84  and  870-899, 960-979, 980-989 |
| Injury or poisoning plus Mental Disorder | At same visit:  881, 960-979, 980-989, 994.7  and  290.8, 290.9, 293.83, 295-299, 300.4, 301, 309, 311, 780.1 |
| **Code Type** | **ICD-10-CM Codes** |
| Suicide Attempt | T14.91, T36.0X, T36.1X, T36.2X, T36.3X, T36.4X, T36.5X, T36.6X, T36.7X, T36.8X, T36.92, T37.0X, T37.1X, T37.2X, T37.3X, T37.4X, T37.5X, T37.8X, T37.92, T38.0X, T38.1X, T38.2X, T38.3X, T38.4X, T38.5X, T38.6X, T38.7X, T38.80, T38.81, T38.89, T38.90, T38.99, T39.01, T39.09, T39.1X, T39.2X, T39.31, T39.39, T39.4X, T39.8X, T39.92, T40.0X, T40.1X, T40.2X, T40.3X, T40.4X, T40.5X, T40.60, T40.69, T40.7X, T40.8X, T40.90, T40.99, T41.0X, T41.1X, T41.20, T41.29, T41.3X, T41.42, T41.5X, T42.0X, T42.1X, T42.2X, T42.3X, T42.4X, T42.5X, T42.6X, T42.72, T42.8X, T43.01, T43.02, T43.1X, T43.20, T43.21, T43.22, T43.29, T43.3X, T43.4X, T43.50, T43.59, T43.60, T43.61, T43.62, T43.63, T43.69, T43.8X, T43.92, T44.0X, T44.1X, T44.2X, T44.3X, T44.4X, T44.5X, T44.6X, T44.7X, T44.8X, T44.90, T44.99, T45.0X, T45.1X, T45.2X, T45.3X, T45.4X, T45.51, T45.52, T45.60, T45.61, T45.62, T45.69, T45.7X, T45.8X, T45.92, T46.0X, T46.1X, T46.2X, T46.3X, T46.4X, T46.5X, T46.6X, T46.7X, T46.8X, T46.90, T46.99, T47.0X, T47.1X, T47.2X, T47.3X, T47.4X, T47.5X, T47.6X, T47.7X, T47.8X, T47.92, T48.0X, T48.1X, T48.20, T48.29, T48.3X, T48.4X, T48.5X, T48.6X, T48.90, T48.99, T49.0X, T49.1X, T49.2X, T49.3X, T49.4X, T49.5X, T49.6X, T49.7X, T49.8X, T49.92, T50.0X, T50.1X, T50.2X, T50.3X, T50.4X, T50.5X, T50.6X, T50.7X, T50.8X, T50.90, T50.99, T50.A1, T50.A2, T50.A9, T50.B1, T50.B9, T50.Z1, T50.Z9, T51.0X, T51.1X, T51.2X, T51.3X, T51.8X, T51.92, T52.0X, T52.1X, T52.2X, T52.3X, T52.4X, T52.8X, T52.92, T53.0X, T53.1X, T53.2X, T53.3X, T53.4X, T53.5X, T53.6X, T53.7X, T53.92, T54.0X, T54.1X, T54.2X, T54.3X, T54.92, T55.0X, T55.1X, T56.0X, T56.1X, T56.2X, T56.3X, T56.4X, T56.5X, T56.6X, T56.7X, T56.81, T56.89, T56.92, T57.0X, T57.1X, T57.2X, T57.3X, T57.8X, T57.92, T58.02, T58.12, T58.2X, T58.8X, T58.92, T59.0X, T59.1X, T59.2X, T59.3X, T59.4X, T59.5X, T59.6X, T59.7X, T59.81, T59.89, T59.92, T60.0X, T60.1X, T60.2X, T60.3X, T60.4X, T60.8X, T60.92, T61.02, T61.12, T61.77, T61.78, T61.8X, T61.92, T62.0X, T62.1X, T62.2X, T62.8X, T62.92, T63.00, T63.01, T63.02, T63.03, T63.04, T63.06, T63.07, T63.08, T63.09, T63.11, T63.12, T63.19, T63.2X, T63.30, T63.31, T63.32, T63.33, T63.39, T63.41, T63.42, T63.43, T63.44, T63.45, T63.46, T63.48, T63.51, T63.59, T63.61, T63.62, T63.63, T63.69, T63.71, T63.79, T63.81, T63.82, T63.83, T63.89, T63.92, T64.02, T64.82, T65.0X, T65.1X, T65.21, T65.22, T65.29, T65.3X, T65.4X, T65.5X, T65.6X, T65.81, T65.82, T65.83, T65.89, T65.92, T71.11, T71.12, T71.13, T71.15, T71.16, T71.19, T71.22, T71.23, X71.0X, X71.1X, X71.2X, X71.3X, X71.8X, X71.9X, X72.XX, X73.0X, X73.1X, X73.2X, X73.8X, X73.9X, X74.01, X74.02, X74.09, X74.8X, X74.9X, X75.XX, X76.XX, X77.0X, X77.1X, X77.2X, X77.3X, X77.8X, X77.9X, X78.0X, X78.1X, X78.2X, X78.8X, X78.9X, X79.XX, X80.XX, X81.0X, X81.1X, X81.8X, X82.0X, X82.1X, X82.2X, X82.8X, X83.0X, X83.1X, X83.2X, X83.8X |
| Suicide Ideation plus Injury or Poisoning | At same visit:  R45.851  and  M1A.10, M1A.11, M1A.12, M1A.13, M1A.14, M1A.15, M1A.16, M1A.17, M1A.18, M1A.19, S01.00, S01.01, S01.02, S01.03, S01.04, S01.05, S01.10, S01.11, S01.12, S01.13, S01.14, S01.15, S01.20, S01.21, S01.22, S01.23, S01.24, S01.25, S01.30, S01.31, S01.32, S01.33, S01.34, S01.35, S01.40, S01.41, S01.42, S01.43, S01.44, S01.45, S01.50, S01.51, S01.52, S01.53, S01.54, S01.55, S01.80, S01.81, S01.82, S01.83, S01.84, S01.85, S01.90, S01.91, S01.92, S01.93, S01.94, S01.95, S02.5X, S03.2X, S05.20, S05.21, S05.22, S05.30, S05.31, S05.32, S05.40, S05.41, S05.42, S05.50, S05.51, S05.52, S05.60, S05.61, S05.62, S05.70, S05.71, S05.72, S05.8X, S05.90, S05.91, S05.92, S08.0X, S08.11, S08.12, S08.81, S08.89, S09.12, S09.20, S09.21, S09.22, S09.30, S09.31, S09.39, S09.8X, S09.90, S09.91, S09.93, S11.01, S11.02, S11.03, S11.10, S11.11, S11.12, S11.13, S11.14, S11.15, S11.20, S11.21, S11.22, S11.23, S11.24, S11.25, S11.80, S11.81, S11.82, S11.83, S11.84, S11.85, S11.89, S11.90, S11.91, S11.92, S11.93, S11.94, S11.95, S16.2X, S21.00, S21.01, S21.02, S21.03, S21.04, S21.05, S21.10, S21.11, S21.12, S21.13, S21.14, S21.15, S21.20, S21.21, S21.22, S21.23, S21.24, S21.25, S21.90, S21.91, S21.92, S21.93, S21.94, S21.95, S28.1X, S28.21, S28.22, S29.02, S31.00, S31.01, S31.02, S31.03, S31.04, S31.05, S31.10, S31.11, S31.12, S31.13, S31.14, S31.15, S31.20, S31.21, S31.22, S31.23, S31.24, S31.25, S31.30, S31.31, S31.32, S31.33, S31.34, S31.35, S31.40, S31.41, S31.42, S31.43, S31.44, S31.45, S31.50, S31.51, S31.52, S31.53, S31.54, S31.55, S31.80, S31.81, S31.82, S31.83, S38.21, S38.22, S38.23, S38.3X, S39.02, S41.00, S41.01, S41.02, S41.03, S41.04, S41.05, S41.10, S41.11, S41.12, S41.13, S41.14, S41.15, S46.02, S46.12, S46.22, S46.32, S46.82, S46.92, S48.01, S48.02, S48.11, S48.12, S48.91, S48.92, S51.00, S51.01, S51.02, S51.03, S51.04, S51.05, S51.80, S51.81, S51.82, S51.83, S51.84, S51.85, S56.02, S56.12, S56.22, S56.32, S56.42, S56.52, S56.82, S56.92, S58.01, S58.02, S58.11, S58.12, S58.91, S58.92, S61.00, S61.01, S61.02, S61.03, S61.04, S61.05, S61.10, S61.11, S61.12, S61.13, S61.14, S61.15, S61.20, S61.21, S61.22, S61.23, S61.24, S61.25, S61.30, S61.31, S61.32, S61.33, S61.34, S61.35, S61.40, S61.41, S61.42, S61.43, S61.44, S61.45, S61.50, S61.51, S61.52, S61.53, S61.54, S61.55, S66.02, S66.12, S66.22, S66.32, S66.42, S66.52, S66.82, S66.92, S68.01, S68.02, S68.11, S68.12, S68.41, S68.42, S68.51, S68.52, S68.61, S68.62, S68.71, S68.72, S71.00, S71.01, S71.02, S71.03, S71.04, S71.05, S71.10, S71.11, S71.12, S71.13, S71.14, S71.15, S76.02, S76.12, S76.22, S76.32, S76.82, S76.92, S78.01, S78.02, S78.11, S78.12, S78.91, S78.92, S81.00, S81.01, S81.02, S81.03, S81.04, S81.05, S81.80, S81.81, S81.82, S81.83, S81.84, S81.85, S86.02, S86.12, S86.22, S86.32, S86.82, S86.92, S88.01, S88.02, S88.11, S88.12, S88.91, S88.92, S91.00, S91.01, S91.02, S91.03, S91.04, S91.05, S91.10, S91.11, S91.12, S91.13, S91.14, S91.15, S91.20, S91.21, S91.22, S91.23, S91.24, S91.25, S91.30, S91.31, S91.32, S91.33, S91.34, S91.35, S96.02, S96.12, S96.22, S96.82, S96.92, S98.01, S98.02, S98.11, S98.12, S98.13, S98.14, S98.21, S98.22, S98.31, S98.32, S98.91, S98.92, T36.0X, T36.1X, T36.2X, T36.3X, T36.4X, T36.5X, T36.6X, T36.7X, T36.8X, T36.91, T36.92, T36.93, T36.94, T37.0X, T37.1X, T37.2X, T37.3X, T37.4X, T37.5X, T37.8X, T37.91, T37.92, T37.93, T37.94, T38.0X, T38.1X, T38.2X, T38.3X, T38.4X, T38.5X, T38.6X, T38.7X, T38.80, T38.81, T38.89, T38.90, T38.99, T39.01, T39.09, T39.1X, T39.2X, T39.31, T39.39, T39.4X, T39.8X, T39.91, T39.92, T39.93, T39.94, T40.0X, T40.1X, T40.2X, T40.3X, T40.4X, T40.5X, T40.60, T40.69, T40.7X, T40.8X, T40.90, T40.99, T41.0X, T41.1X, T41.20, T41.29, T41.3X, T41.41, T41.42, T41.43, T41.44, T41.5X, T42.0X, T42.1X, T42.2X, T42.3X, T42.4X, T42.5X, T42.6X, T42.71, T42.72, T42.73, T42.74, T42.8X, T43.01, T43.02, T43.1X, T43.20, T43.21, T43.22, T43.29, T43.3X, T43.4X, T43.50, T43.59, T43.60, T43.61, T43.62, T43.63, T43.69, T43.8X, T43.91, T43.92, T43.93, T43.94, T44.0X, T44.1X, T44.2X, T44.3X, T44.4X, T44.5X, T44.6X, T44.7X, T44.8X, T44.90, T44.99, T45.0X, T45.1X, T45.2X, T45.3X, T45.4X, T45.51, T45.52, T45.60, T45.61, T45.62, T45.69, T45.7X, T45.8X, T45.91, T45.92, T45.93, T45.94, T46.0X, T46.1X, T46.2X, T46.3X, T46.4X, T46.5X, T46.6X, T46.7X, T46.8X, T46.90, T46.99, T47.0X, T47.1X, T47.2X, T47.3X, T47.4X, T47.5X, T47.6X, T47.7X, T47.8X, T47.91, T47.92, T47.93, T47.94, T48.0X, T48.1X, T48.20, T48.29, T48.3X, T48.4X, T48.5X, T48.6X, T48.90, T48.99, T49.0X, T49.1X, T49.2X, T49.3X, T49.4X, T49.5X, T49.6X, T49.7X, T49.8X, T49.91, T49.92, T49.93, T49.94, T50.0X, T50.1X, T50.2X, T50.3X, T50.4X, T50.5X, T50.6X, T50.7X, T50.8X, T50.90, T50.99, T50.A1, T50.A2, T50.A9, T50.B1, T50.B9, T50.Z1, T50.Z9, T51.0X, T51.1X, T51.2X, T51.3X, T51.8X, T51.91, T51.92, T51.93, T51.94, T52.0X, T52.1X, T52.2X, T52.3X, T52.4X, T52.8X, T52.91, T52.92, T52.93, T52.94, T53.0X, T53.1X, T53.2X, T53.3X, T53.4X, T53.5X, T53.6X, T53.7X, T53.91, T53.92, T53.93, T53.94, T54.0X, T54.1X, T54.2X, T54.3X, T54.91, T54.92, T54.93, T54.94, T55.0X, T55.1X, T56.0X, T56.1X, T56.2X, T56.3X, T56.4X, T56.5X, T56.6X, T56.7X, T56.81, T56.89, T56.91, T56.92, T56.93, T56.94, T57.0X, T57.1X, T57.2X, T57.3X, T57.8X, T57.91, T57.92, T57.93, T57.94, T58.01, T58.02, T58.03, T58.04, T58.11, T58.12, T58.13, T58.14, T58.2X, T58.8X, T58.91, T58.92, T58.93, T58.94, T59.0X, T59.1X, T59.2X, T59.3X, T59.4X, T59.5X, T59.6X, T59.7X, T59.81, T59.89, T59.91, T59.92, T59.93, T59.94, T60.0X, T60.1X, T60.2X, T60.3X, T60.4X, T60.8X, T60.91, T60.92, T60.93, T60.94, T61.01, T61.02, T61.03, T61.04, T61.11, T61.12, T61.13, T61.14, T61.77, T61.78, T61.8X, T61.91, T61.92, T61.93, T61.94, T62.0X, T62.1X, T62.2X, T62.8X, T62.91, T62.92, T62.93, T62.94, T63.00, T63.01, T63.02, T63.03, T63.04, T63.06, T63.07, T63.08, T63.09, T63.11, T63.12, T63.19, T63.2X, T63.30, T63.31, T63.32, T63.33, T63.39, T63.41, T63.42, T63.43, T63.44, T63.45, T63.46, T63.48, T63.51, T63.59, T63.61, T63.62, T63.63, T63.69, T63.71, T63.79, T63.81, T63.82, T63.83, T63.89, T63.91, T63.92, T63.93, T63.94, T64.01, T64.02, T64.03, T64.04, T64.81, T64.82, T64.83, T64.84, T65.0X, T65.1X, T65.21, T65.22, T65.29, T65.3X, T65.4X, T65.5X, T65.6X, T65.81, T65.82, T65.83, T65.89, T65.91, T65.92, T65.93, T65.94, T78.1X |
| Injury or Poisoning plus Mental Disorder | At same visit:  M1A.10, M1A.11, M1A.12, M1A.13, M1A.14, M1A.15, M1A.16, M1A.17, M1A.18, M1A.19, S51.80, S51.81, S51.83, S51.85, T36.0X, T36.1X, T36.2X, T36.3X, T36.4X, T36.5X, T36.6X, T36.7X, T36.8X, T36.91, T36.92, T36.93, T36.94, T37.0X, T37.1X, T37.2X, T37.3X, T37.4X, T37.5X, T37.8X, T37.91, T37.92, T37.93, T37.94, T38.0X, T38.1X, T38.2X, T38.3X, T38.4X, T38.5X, T38.6X, T38.7X, T38.80, T38.81, T38.89, T38.90, T38.99, T39.01, T39.09, T39.1X, T39.2X, T39.31, T39.39, T39.4X, T39.8X, T39.91, T39.92, T39.93, T39.94, T40.0X, T40.1X, T40.2X, T40.3X, T40.4X, T40.5X, T40.60, T40.69, T40.7X, T40.8X, T40.90, T40.99, T41.0X, T41.1X, T41.20, T41.29, T41.3X, T41.41, T41.42, T41.43, T41.44, T41.5X, T42.0X, T42.1X, T42.2X, T42.3X, T42.4X, T42.5X, T42.6X, T42.71, T42.72, T42.73, T42.74, T42.8X, T43.01, T43.02, T43.1X, T43.20, T43.21, T43.22, T43.29, T43.3X, T43.4X, T43.50, T43.59, T43.60, T43.61, T43.62, T43.63, T43.69, T43.8X, T43.91, T43.92, T43.93, T43.94, T44.0X, T44.1X, T44.2X, T44.3X, T44.4X, T44.5X, T44.6X, T44.7X, T44.8X, T44.90, T44.99, T45.0X, T45.1X, T45.2X, T45.3X, T45.4X, T45.51, T45.52, T45.60, T45.61, T45.62, T45.69, T45.7X, T45.8X, T45.91, T45.92, T45.93, T45.94, T46.0X, T46.1X, T46.2X, T46.3X, T46.4X, T46.5X, T46.6X, T46.7X, T46.8X, T46.90, T46.99, T47.0X, T47.1X, T47.2X, T47.3X, T47.4X, T47.5X, T47.6X, T47.7X, T47.8X, T47.91, T47.92, T47.93, T47.94, T48.0X, T48.1X, T48.20, T48.29, T48.3X, T48.4X, T48.5X, T48.6X, T48.90, T48.99, T49.0X, T49.1X, T49.2X, T49.3X, T49.4X, T49.5X, T49.6X, T49.7X, T49.8X, T49.91, T49.92, T49.93, T49.94, T50.0X, T50.1X, T50.2X, T50.3X, T50.4X, T50.5X, T50.6X, T50.7X, T50.8X, T50.90, T50.99, T50.A1, T50.A2, T50.A9, T50.B1, T50.B9, T50.Z1, T50.Z9, T51.0X, T51.1X, T51.2X, T51.3X, T51.8X, T51.91, T51.92, T51.93, T51.94, T52.0X, T52.1X, T52.2X, T52.3X, T52.4X, T52.8X, T52.91, T52.92, T52.93, T52.94, T53.0X, T53.1X, T53.2X, T53.3X, T53.4X, T53.5X, T53.6X, T53.7X, T53.91, T53.92, T53.93, T53.94, T54.0X, T54.1X, T54.2X, T54.3X, T54.91, T54.92, T54.93, T54.94, T55.0X, T55.1X, T56.0X, T56.1X, T56.2X, T56.3X, T56.4X, T56.5X, T56.6X, T56.7X, T56.81, T56.89, T56.91, T56.92, T56.93, T56.94, T57.0X, T57.1X, T57.2X, T57.3X, T57.8X, T57.91, T57.92, T57.93, T57.94, T58.01, T58.02, T58.03, T58.04, T58.11, T58.12, T58.13, T58.14, T58.2X, T58.8X, T58.91, T58.92, T58.93, T58.94, T59.0X, T59.1X, T59.2X, T59.3X, T59.4X, T59.5X, T59.6X, T59.7X, T59.81, T59.89, T59.91, T59.92, T59.93, T59.94, T60.0X, T60.1X, T60.2X, T60.3X, T60.4X, T60.8X, T60.91, T60.92, T60.93, T60.94, T61.01, T61.02, T61.03, T61.04, T61.11, T61.12, T61.13, T61.14, T61.77, T61.78, T61.8X, T61.91, T61.92, T61.93, T61.94, T62.0X, T62.1X, T62.2X, T62.8X, T62.91, T62.92, T62.93, T62.94, T63.00, T63.01, T63.02, T63.03, T63.04, T63.06, T63.07, T63.08, T63.09, T63.11, T63.12, T63.19, T63.2X, T63.30, T63.31, T63.32, T63.33, T63.39, T63.41, T63.42, T63.43, T63.44, T63.45, T63.46, T63.48, T63.51, T63.59, T63.61, T63.62, T63.63, T63.69, T63.71, T63.79, T63.81, T63.82, T63.83, T63.89, T63.91, T63.92, T63.93, T63.94, T64.01, T64.02, T64.03, T64.04, T64.81, T64.82, T64.83, T64.84, T65.0X, T65.1X, T65.21, T65.22, T65.29, T65.3X, T65.4X, T65.5X, T65.6X, T65.81, T65.82, T65.83, T65.89, T65.91, T65.92, T65.93, T65.94, T71.11, T71.12, T71.13, T71.14, T71.15, T71.16, T71.19, T71.20, T71.21, T71.22, T71.23, T71.29, T71.9X, T78.1X, Y21.9X, Y22.XX, Y23.0X, Y23.1X, Y23.2X, Y23.3X, Y23.8X, Y23.9X, Y24.0X, Y24.8X, Y24.9X, Y25.XX, Y26.XX, Y27.0X, Y27.1X, Y27.2X, Y27.3X, Y27.8X, Y27.9X, Y28.0X, Y28.1X, Y28.2X, Y28.8X, Y28.9X, Y29.XX, Y30.XX, Y31.XX, Y32.XX, Y33.XX  and  F03.90, F06.30, F06.31, F06.32, F06.33, F06.34, F20.89, F21., F22., F23., F28., F29., F30.10, F30.11, F30.12, F30.13, F30.2, F30.3, F30.4, F30.8, F30.9, F31.0, F31.10, F31.11, F31.12, F31.13, F31.2, F31.30, F31.31, F31.32, F31.4, F31.5, F31.60, F31.61, F31.62, F31.63, F31.64, F31.70, F31.71, F31.72, F31.73, F31.74, F31.75, F31.76, F31.77, F31.78, F31.81, F31.89, F31.9, F32.0, F32.1, F32.2, F32.3, F32.4, F32.5, F32.89, F32.9, F33.0, F33.1, F33.2, F33.3, F33.40, F33.41, F33.42, F33.8, F33.9, F34.0, F34.1, F34.81, F34.89, F34.9, F43.10, F43.11, F43.12, F43.20, F43.21, F43.22, F43.23, F43.24, F43.25, F43.29, F43.8, F43.9, F44.89, F60.0, F60.1, F84.0, F93.0, F94.8, R44.0, R44.2, R44.3 |

**Supplemental Table 2**: ICD-9 and ICD-10 clinical modification (CM) diagnosis codes used for identification of traumatic brain injury and posttraumatic stress disorder.

|  | **ICD-9-CM Codes** | **ICD-10-CM Codes** |
| --- | --- | --- |
| **Traumatic Brain Injury (TBI)** | 310.2, 800.xx, 801.xx, 803.xx, 804.xx, 850.xx, 852.xx, 853.xx, 854.xx, 905.0, 907.0, 950.1, 950.2, 950.3, 959.01, 959.9, V15.52 | F07.81, S02.0xxx, S02.1xxx, S02.8xxx, S02.9xxx, S04.02xx, S04.03xx, S04.04xx, S06.0xxx, S06.1xxx, S06.2xxx, S06.3xxx, S06.4xxx, S06.5xxx, S06.6xxx, S06.8xxx, S06.9xxx, S07.1xxx, Z87.820 |
| **Posttraumatic Stress Disorder (PTSD)** | 309.81 | F43.10-F43.12 |

**Supplemental Table 3**: Odds of intentional self-harm by Veterans’ TBI and PTSD diagnosis status and Veteran characteristics, stratified by VHA utilization.

|  |  | **Logistic Regression** | |
| --- | --- | --- | --- |
|  | Bivariable | Multi-variable | |
| Veteran Characteristics | Model | Minimally Adjusted Model* | Fully Adjusted Model** |
| *Low Healthcare use (less than 50th percentile)* | OR (95% CI) | OR (95% CI) | OR (95% CI) |
| **TBI and PTSD Diagnoses** |  |  |  |
| Neither | ref. | ref. | ref. |
| TBI-only | 3.79 [3.31, 4.34] | 2.60 [2.27, 2.98] | 2.48 [2.17, 2.84] |
| PTSD-only | 7.33 [7.04, 7.64] | 5.22 [5.01, 5.44] | 5.14 [4.93, 5.35] |
| Comorbid TBI-PTSD | 9.05 [8.10, 10.11] | 4.85 [4.34, 5.42] | 4.83 [4.32, 5.40] |
| **Age (Years)** |  |  |  |
| < 30 | 2.69 [2.57, 2.82] | 2.13 [2.03, 2.23] | 2.41 [2.30, 2.52] |
| 30 - 44 | 1.57 [1.51, 1.65] | 1.33 [1.27, 1.39] | 1.56 [1.49, 1.63] |
| 45 - 69 | ref. | ref. | ref. |
| >= 70 | 0.17 [0.16, 0.19] | 0.21 [0.20, 0.23] | 0.20 [0.18, 0.22] |
| **Sex** |  |  |  |
| Female | 1.56 [1.46, 1.67] | 1.08 [1.01, 1.15] | 1.03 [0.96, 1.10] |
| Male | ref. | ref. | ref. |
| **Race** |  |  |  |
| White | ref. | ref. | ref. |
| Black or African American | 1.26 [1.19, 1.32] | 0.97 [0.92, 1.02] | 0.88 [0.84, 0.93] |
| Asian | 1.36 [1.17, 1.59] | 1.04 [0.89, 1.21] | 1.08 [0.93, 1.26] |
| American Indian or Alaskan Native | 1.51 [1.27, 1.80] | 1.05 [0.88, 1.25] | 1.02 [0.85, 1.21] |
| Native Hawaiian or Other Pacific Islander | 1.47 [1.24, 1.74] | 1.15 [0.98, 1.37] | 1.19 [1.00, 1.41] |
| Other | 1.20 [1.10, 1.31] | 1.04 [0.95, 1.13] | 1.12 [1.02, 1.22] |
| **Ethnicity** |  |  |  |
| Hispanic | 1.26 [1.16, 1.36] | - | 0.84 [0.78, 0.90] |
| Not Hispanic | ref. | - | ref. |
| **Marital Status** |  |  |  |
| Single | 2.89 [2.76, 3.03] | - | 1.69 [1.62, 1.77] |
| Married | ref. | - | ref. |
| Widowed | 0.75 [0.68, 0.83] | - | 1.60 [1.44, 1.78] |
| Divorced | 3.01 [2.88, 3.12] | - | 2.33 [2.24, 2.42] |
| Unknown | 1.46 [1.18, 1.82] | - | 1.17 [0.94, 1.46] |
| **VA Service Connection Status** |  |  |  |
| No Service Connection | ref. | - | ref. |
| Service Connection < 50% | 0.96 [0.91, 1.01] | - | 0.72 [0.68, 0.76] |
| Service Connection >= 50% | 2.10 [2.02, 2.19] | - | 0.91 [0.88, 0.95] |
| **Charlson Comorbidity Index** |  |  |  |
| < 5 | ref. | - | ref. |
| 5 - 15 | 0.60 [0.58, 0.63] | - | 1.49 [1.43, 1.56] |
| > 15 | 1.35 [1.06, 1.72] | - | 2.62 [2.06, 3.34] |
| *Medium Healthcare use (50th - 75th percentile)* | OR (95% CI) | OR (95% CI) | OR (95% CI) |
| **TBI and PTSD Diagnoses** |  |  |  |
| Neither | ref. | ref. | ref. |
| TBI-only | 3.54 [3.25, 3.85] | 2.33 [2.14, 2.54] | 2.32 [2.13, 2.53] |
| ­PTSD-only | 4.68 [4.54, 4.82] | 2.91 [2.83, 3.00] | 3.23 [3.14, 3.33] |
| Comorbid TBI-PTSD | 7.42 [7.01, 7.85] | 2.81 [2.65, 2.97] | 3.17 [2.99, 3.36] |
| **Age (Years)** |  |  |  |
| < 30 | 5.24 [5.03, 5.46] | 3.53 [3.39, 3.67] | 4.21 [4.04, 4.38] |
| 30 - 44 | 2.30 [2.23, 2.38] | 1.80 [1.74, 1.86] | 2.20 [2.13, 2.28] |
| 45 - 69 | ref. | ref. | ref. |
| >= 70 | 0.18 [0.17, 0.19] | 0.22 [0.21, 0.23] | 0.20 [0.19, 0.21] |
| **Sex** |  |  |  |
| Female | 1.74 [1.66, 1.82] | 1.04 [1, 1.09] | 1.01 [0.96, 1.05] |
| Male | ref. | ref. | ref. |
| **Race** |  |  |  |
| White | ref. | ref. | ref. |
| Black or African American | 1.07 [1.03, 1.11] | 0.86 [0.83, 0.89] | 0.81 [0.78, 0.84] |
| Asian | 1.12 [0.96, 1.30] | 0.76 [0.66, 0.89] | 0.83 [0.71, 0.96] |
| American Indian or Alaskan Native | 1.56 [1.37, 1.78] | 1.07 [0.94, 1.22] | 1.05 [0.92, 1.20] |
| Native Hawaiian or Other Pacific Islander | 1.44 [1.27, 1.63] | 1.17 [1.03, 1.33] | 1.22 [1.08, 1.38] |
| Other | 0.97 [0.91, 1.04] | 0.87 [0.81, 0.93] | 0.90 [0.84, 0.96] |
| **Ethnicity** |  |  |  |
| Hispanic | 1.21 [1.14, 1.28] | - | 0.85 [0.80, 0.90] |
| Not Hispanic | ref. | - | ref. |
| **Marital Status** |  |  |  |
| Single | 2.92 [2.81, 3.03] | - | 1.62 [1.56, 1.68] |
| Married | ref. | - | ref. |
| Widowed | 0.78 [0.73, 0.84] | - | 1.66 [1.55, 1.79] |
| Divorced | 2.59 [2.51, 2.67] | - | 2.13 [2.07, 2.19] |
| Unknown | 2.79 [2.25, 3.46] | - | 1.80 [1.45, 2.23] |
| **VA Service Connection Status** |  |  |  |
| No Service Connection | ref. | - | ref. |
| Service Connection < 50% | 1.08 [1.04, 1.13] | - | 0.73 [0.70, 0.76] |
| Service Connection >= 50% | 2.07 [2.01, 2.13] | - | 0.77 [0.75, 0.79] |
| **Charlson Comorbidity Index** |  |  |  |
| < 5 | ref. | - | ref. |
| 5 - 15 | 0.50 [0.48, 0.51] | - | 1.42 [1.38, 1.47] |
| > 15 | 0.66 [0.57, 0.77] | - | 2.01 [1.73, 2.34] |
| *High Healthcare use (75th percentile and higher)* | OR (95% CI) | OR (95% CI) | OR (95% CI) |
| **TBI and PTSD Diagnoses** |  |  |  |
| Neither | ref. | ref. | ref. |
| TBI-only | 2.81 [2.69, 2.93] | 2.50 [2.40, 2.61] | 2.44 [2.34, 2.55] |
| PTSD-only | 3.21 [3.16, 3.26] | 2.35 [2.31, 2.39] | 2.90 [2.85, 2.95] |
| Comorbid TBI-PTSD | 6.72 [6.54, 6.90] | 3.40 [3.31, 3.49] | 4.26 [4.15, 4.38] |
| **Age (Years)** |  |  |  |
| < 30 | 4.99 [4.83, 5.17] | 3.29 [3.18, 3.41] | 3.78 [3.65, 3.92] |
| 30 - 44 | 2.36 [2.31, 2.41] | 1.80 [1.76, 1.84] | 2.15 [2.10, 2.19] |
| 45 - 69 | ref. | ref. | ref. |
| >= 70 | 0.24 [0.23, 0.25] | 0.30 [0.29, 0.30] | 0.29 [0.28, 0.30] |
| **Sex** |  |  |  |
| Female | 1.56 [1.52, 1.60] | 1.05 [1.02, 1.07] | 1.00 [0.97, 1.03] |
| Male | ref. | ref. | ref. |
| **Race** |  |  |  |
| White | ref. | ref. | ref. |
| Black or African American | 0.97 [0.95, 0.99] | 0.87 [0.85, 0.89] | 0.81 [0.79, 0.83] |
| Asian | 1.03 [0.93, 1.14] | 0.75 [0.67, 0.83] | 0.81 [0.73, 0.90] |
| American Indian or Alaskan Native | 1.48 [1.37, 1.60] | 1.10 [1.02, 1.18] | 1.09 [1.01, 1.17] |
| Native Hawaiian or Other Pacific Islander | 0.88 [0.81, 0.97] | 0.77 [0.70, 0.84] | 0.81 [0.74, 0.89] |
| Other | 0.85 [0.82, 0.89] | 0.80 [0.76, 0.83] | 0.83 [0.80, 0.87] |
| **Ethnicity** |  |  |  |
| Hispanic | 1.05 [1.02, 1.09] | - | 0.89 [0.86, 0.92] |
| Not Hispanic | ref. | - | ref. |
| **Marital Status** |  |  |  |
| Single | 2.73 [2.67, 2.79] | - | 1.93 [1.89, 1.97] |
| Married | ref. | - | ref. |
| Widowed | 0.97 [0.94, 1.01] | - | 1.69 [1.62, 1.75] |
| Divorced | 2.36 [2.32, 2.40] | - | 2.08 [2.05, 2.12] |
| Unknown | 1.70 [1.37, 2.13] | - | 1.27 [1.02, 1.58] |
| **VA Service Connection Status** |  |  |  |
| No Service Connection | ref. | - | ref. |
| Service Connection < 50% | 1.08 [1.05, 1.10] | - | 0.83 [0.81, 0.85] |
| Service Connection >= 50% | 1.43 [1.41, 1.46] | - | 0.66 [0.65, 0.67] |
| **Charlson Comorbidity Index** |  |  |  |
| < 5 | ref. | - | ref. |
| 5 - 15 | 0.57 [0.56, 0.58] | - | 1.29 [1.27, 1.31] |
| > 15 | 0.56 [0.53, 0.59] | - | 1.61 [1.53, 1.70] |

*Minimally adjusted model included age, sex, and race.

**Fully adjusted model included age, sex, race, ethnicity, marital status, VA service-connection status, and Charlson Comorbidity Index scores.

*Note*: For each demographic variable, the largest group is chosen as the referent for both models.
